# Simultaneous identification of the *Anopheles funestus *group and *Anopheles longipalpis *type C by PCR-RFLP

**DOI:** 10.1186/1475-2875-9-316

**Published:** 2010-11-08

**Authors:** Kwang Shik Choi, Maureen Coetzee, Lizette L Koekemoer

**Affiliations:** 1 Vector Control Reference Unit, National Institute for Communicable Diseases, National Health Laboratory Service, Johannesburg, South Africa; 2 Malaria Entomology Research Unit, School of Pathology, Faculty of Health Sciences, University of the Witwatersrand, Johannesburg, South Africa

## Abstract

**Background:**

*Anopheles longipalpis *is morphologically similar to the major African malaria vector *Anopheles funestus *at the adult stage although it is very different at the larval stage. Despite the development of the species-specific multiplex PCR assay for the *An. funestus *group, the genomic DNA of *Anopheles longipalpis *type C specimens can be amplified with the *Anopheles vaneedeni *and *Anopheles parensis *primers from this assay. The standard, species-specific *An. funestus *group PCR, results in the amplification of two fragments when *An. longipalpis *type C specimens are included in the analysis. This result can easily be misinterpreted as being a hybrid between *An. vaneedeni *and *An. parensis*. *Anopheles longipalpis *type C can be identified using a species-specific PCR assay but this assay is not reliable if other members of the *An. funestus *group, such as *An. funestus*, *An. funestus*-like and *An. parensis*, are included. The present study provides a multiplex assay that will identify *An. longipalpis *along with other common members of the African *An. funestus *group, including *Anopheles leesoni*.

**Methods:**

A total of 70 specimens from six species (*An. funestus*, *An. funestus*-like, *An. parensis*, *Anopheles rivulorum*, *An. vaneedeni *and *An. leesoni*) in the *An. funestus *group and *An. longipalpis *type C from Malawi, Mozambique, South Africa and Zambia were used for the study. A restriction fragment length polymorphism (RFLP) assay was designed based on the DNA sequence information in the GenBank database.

**Results:**

The enzyme, *EcoRI *digested only *An. longipalpis *type C and *An. funestus*-like after the species-specific *An. funestus *group PCR assay. The *An. longipalpis *and *An. funestus*-like digestion profiles were characterized by three fragments, 376 bp, 252 bp and 211 bp for *An. longipalpis *type C and two fragments, 375 bp and 15 bp for *An. funestus*-like.

**Conclusions:**

An RFLP method for the group was developed that is more accurate and efficient than those used before. Hence, this assay would be useful for field-collected adult specimens to be identified routinely in malaria vector research and control studies.

## Background

The *Anopheles funestus *group originally consisted of nine species [1,2]: the major African malaria vector *An. funestus *and eight minor or non-vectors, *Anopheles aruni*, *Anopheles parensis*, *Anopheles vaneedeni*, *Anopheles confusus*, *Anopheles rivulorum*, *Anopheles leesoni*, *Anopheles brucei *and *Anopheles fuscivenosus*. Subsequent studies on the systematics of the group have resulted in a reclassification of the group with *An. funestus*, *An. aruni*, *An. parensis*, *An. confusus *and *An. vaneedeni *being grouped together as members of the "*An. funestus *subgroup", *An. rivulorum*, *An. rivulorum*-like, *An. brucei *and *An. fuscivenosus *forming their own subgroup, and *An. leesoni *being classified with the Asian *Anopheles minimus *subgroup [3]. Recently, a new species has been described from Malawi, *An. funestus*-like [4], and this species falls within the *An. funestus *subgroup.

In addition to the 10 species mentioned above, there are closely related species that are not included in the group because of morphological differences in the adult females - *Anopheles longipalpis *being one of these [1]. These differences are very subtle and the probability of confusing this species with the vector *An. funestus *is very high. To further complicate the issue, molecular studies on this taxon have revealed at least two cryptic species, one from South Africa (Type A) and the other from Zambia (Type C) [5].

The adult biology of *An. longipalpis *has not been well studied despite the fact that it is widely distributed in eastern and southern Africa, from Sudan to Angola and South Africa [1,2,6]. Smith [7,8] reported human feeding behaviour in *An. longipalpis *from indoor and outdoor collections on Ukara Island in Lake Victoria and outdoors in Tanzania. Adugna and Petros [9] also found *An. longipalpis *specimens containing human blood meals from their collections in Ethiopia. Recently, Kent *et al *[10] reported that although the species is found in large numbers resting indoors in sympatry with *An. funestus *and *Anopheles arabiensis *in Zambia, it is predominantly zoophilic with only a small number feeding on humans. The species has never been implicated as a vector of malaria or involved in malaria transmission [1,2,10,11]. However, it may be a secondary vector of malaria in areas of sufficiently high endemicity and densities of this species [10].

Although adult *An. longipalpis *are morphologically characterized by maxillary palpal length and pale basal and apical bands spanning the hind tarsal joints [1,2], this species can be misidentified as *An. funestus *due to similarities of wing venation, palpal banding patterns and small body size. Currently, there are two identification methods for field-collected specimens of *An. longipalpis*. The first method, suggested by Kent *et al *[10], is to identify *An. longipalpis *using the species-specific PCR assay for the *An. funestus *group from the internal transcribed spacer (ITS) region of the rDNA [11]. The assay produces two diagnostic fragments from *An. longipalpis *type C which correlates with *An. parensis *and *An. vaneedeni *due to sequence similarity among these species. However, this analysis would lead to misidentification with hybrids of *An. parensis *and *An. vaneedeni*, although these have never been found in nature. The second method is a species-specific PCR assay for *An. longipalpis *from the ITS2 region developed by Koekemoer *et al *[5], but this is not specific when other members of the *An. funestus *group are included such as *An. funestus*, *An. funestus*-like and *An. parensis*. Hence, this study proposes a new method for the molecular identification of *An. longipalpis *type C and six members of the *An. funestus *group using a Restriction Fragment Length Polymorphism (RFLP) that is more accurate and efficient than currently used methods.

## Methods

### Mosquito samples collection and identification

Mosquitoes were collected from Malawi, Mozambique, South Africa and Zambia (Table 1). There were eleven *An. longipalpis *type C specimen available. Initially, specimens were identified morphologically using the keys of Gillies and Coetzee [2]. All DNA samples were extracted from either single mosquitoes or available parts of mosquitoes using the Ballinger-Crabtree protocol [12] except *An. longipalpis *where the method of Collins *et al *[13] was used. The DNA templates were resuspended in TE buffer at volumes between 50 μL and 100 μL. All DNA templates were identified using the method of Spillings *et al *[4] for *An. funestus*-like and the method of Koekemoer *et al *[11] for the rest of the species.

**Table 1:** List of species, localities and numbers used.

Species	Localities		Numbers used	Total
	Country	Village		
*An. funestus*	Mozambique	Chibuto (24°40'S, 33°33'E)	3	5
	Malawi	Karonga (10°19'S, 34°08'E)	2	
*An. funestus*-like	Malawi	Karonga (10°19'S, 34°08'E)	5	5
*An. leesoni*	South Africa	Komatipoort (25°26'S, 31°57'E)	3	3
*An. longipalpis *type C	Zambia	Macha (16°46'S, 26°94'E)	11	11
*An. parensis*	South Africa	Mamfene (27°23'S, 32°12'E)	5	5
*An. rivulorum*	South Africa	Komatipoort (25°26'S, 31°57'E)	5	5
		Giyani area (23°15'S, 30°47'E)	7	
*An. vaneedeni*	South Africa	Komatipoort area (25°26'S, 31°57'E)	28	46
		Mamfene area (27°23'S, 32°12'E)	10	
		Ndumu (27°02'S, 32°19'E)	1	

### Restriction fragment length polymorphism (RFLP) assay

Although the method of Spillings *et al *[4] can be carried out simultaneously with the method of Koekemoer *et al *[11], they recommend that these assays be carried out separately because the *An. funestus*-like 390 bp fragment of Spillings *et al *[4] is close to the *An. rivulorum *411 bp fragment of Koekemoer *et al *[11]. The procedure in the present study simultaneously carried out both these methods [4,11] with minor modifications. The G^AATTC restriction site for *EcoRI *enzyme lies within the *An. funestus*-like and *An. longipalpis *type C fragments amplified by the methods of Spillings *et al *[4] and Koekemoer *et al *[11] respectively. The restriction enzyme digested only the fragments for *An. funestus*-like and *An. longipalpis *type C directly. A total volume of 25 μL for each PCR reaction contained 1 μL of the genomic DNA of an individual mosquito, 1 × PCR Buffer, 0.2 mM of each dNTP, 0.26 pM (0.2 μM of MalaFB primer) of each primer, and 1 unit of *Taq *DNA polymerase. The PCR cycling conditions were as follows: a 2 minute 94°C followed by 35 cycles of 30 seconds at 94°C, 30 seconds at 50°C and 40 seconds at 72°C; there was a final extension step of 10 minutes at 72°C. After amplification, 1 unit of *EcoRI *in 1 × buffer *EcoRI *(Roche Diagnostic) was added to the PCR reactions and digestion carried out at 37°C for a minimum of three hours. Digested fragments were electrophoresed through an ethidium bromide 2.5% agarose gel and photographed under ultraviolet light illumination using a gel imaging system.

## Results

**Figure 1:**
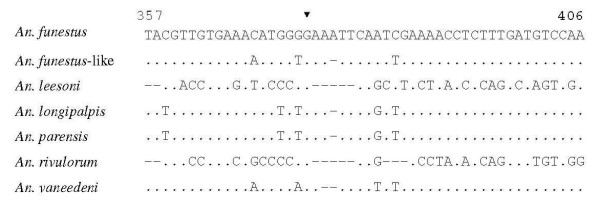
**Alignment from 3' to 5' end of the ITS2 region of the rDNA sequenced by Koekemoer *et al ***[5]**for *An. longipalpis *type C, Spillings *et al ***[4]**for *An. funestus*-like and Koekemoer *et al ***[11]**for the rest of the species**. Dots represent identity with respect to the *An. funestus *sequences. Dashes represent gaps in sequences. The black triangle indicates the restriction site of the *EcoRI *enzyme (G^AATTC) in *An. funestus*-like and *An. longipalpis *type C. The amplification for *An. parensis *after PCR-RFLP was not included in these sites.

**Figure 2:**
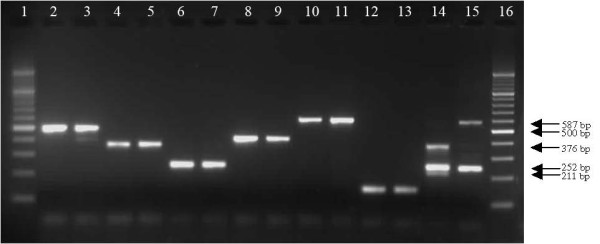
**An ethidium bromide stained 2.5% agarose gel showing the DNA bands after PCR-RFLP**. Lanes 1 and 16 = 100 bp molecular marker; lanes 2 and 3 = *An. funestus*; lanes 4 and 5 = *An. funestus*-like; lanes 6 and 7 = *An. parensis*; lanes 8 and 9 = *An. rivulorum*; lanes 10 and 11 = *An. vaneedeni*; lanes 12 and 13 = *An. leesoni*; lane 14 = *An. longipalpis *type C; lane 15 = 1 : 1 *An. parensis *and *An. vaneedeni *mixed DNAs.

The PCR-RFLP assay for the six members of the *An. funestus *group and *An. longipalpis *type C, resulted in different sizes of DNA fragments as recorded in Table 2. Two fragments (252 bp and 587 bp) of *An. longipalpis *type C were amplified after the species-specific *An. funestus *group PCR. The restriction enzyme digested only the fragments for *An. funestus*-like and *An. longipalpis *type C. There were restriction sites at position 375 in *An. funestus*-like and at position 376 in the large fragment of *An. longipalpis *type C (Figure 1). Although the restriction site was identical to the DNA sequence of *An. parensis*, the specific fragment was not involved in the primer design for the method of Koekemoer *et al *[11]. The digestion profiles for *An. funestus*-like and *An. longipalpis *type C were characterized by two fragments, 375 bp and 15 bp and three fragments, 211 bp, 252 bp and 376 bp respectively. However, the length of fragment for *An. funestus*-like after PCR amplification was not accurate due to approximate length for the species in Spillings *et al *[4] and not available for the full alignment of DNA sequences in GenBank. Figure 2 shows DNA bands for the six members of the *An. funestus *group and *An. longipalpis *type C produced after PCR amplification and restriction enzyme digestion.

**Table 2:** Sizes of the DNA fragments after PCR-RFLP for the *An. funestus *group and *An. longipalpis*.

Species	PCR product sizes (bp)	DNA fragment length after digestion with *EcoRI *(bp)		
*An. funestus*	505	505		
*An. funestus*-like	390 (approx.)	375	15	
*An. leesoni*	146	146		
*An. Longipalpis *type C	587, 252	376	252	211
*An. parensis*	252	252		
*An. rivulorum*	411	411		
*An. vaneedeni*	587	587		

## Discussion

The assay for the *An. funestus *group and *An. longipalpis *type C used in this study requires one PCR and one RFLP step instead of the three PCR steps currently needed. This will save time and reagents when routinely identifying collections of wild mosquitoes belonging to this important group. All seven species were easily separated on a 2.5% ethidium bromide agarose gel even though the digested amplicons of *An. longipalpis *type C (252 bp and 211 bp), *An. funestus*-like (375 bp) and *An. rivulorum *(411 bp) are close to each other. However, individuals that do not amplify at all may need additional processing using the *An. longipalpis *type A primers [5].

Koekemoer *et al *[5] reported that sequence analysis of the ITS2 region in *An. longipalpis *type C is similar to the sequences of *An. parensis *and *An. vaneedeni *in the *An. funestus *subgroup. These three species are almost identical at the adult stage with only a few minor differences [1,2]. However, *An. longipalpis *was not included in the *An. funestus *group because of its very different larval morphology [1,3]. Koekemoer *et al *[5] indicated that it might be due to extensive evolutionary divergence and the sequence differentiation for *An. longipalpis *type A, which is close to *Anopheles pampanai *and *Anopheles varuna *in the Asian *An. minimus *group based on the DNA analysis of the ITS2 region. They suggested that *An. longipalpis *type C should be placed in the *An. funestus *group supported by sequence similarity to both *An. parensis *and *An. vaneedeni*. Furthermore, Kent *et al*. [10] also reported that the fragments diagnostic for *An. longipalpis *type C were the same as the diagnostic amplicons for a hypothetical *An. parensis*/*An. vaneedeni *hybrid although no such hybrids have been recorded in nature. However, further investigation of *An. longipalpis *is still required with more field specimens.

## Conclusion

The application of the method described is expected to greatly improve the efficiency of large-scale analysis of field-collected samples of the *An. funestus *group and *An. longipalpis *type C.

## Conflict of interests

The authors declare that they have no competing interests.

## Authors' contributions

KSC designed the study, developed the new RFLP assay and drafted the manuscript. MC and LLK assisted with analysis of the data and helped draft the manuscript.

All authors have read and approved the final manuscript.
